# Health by Design: Interweaving Health Promotion into Environments and Settings

**DOI:** 10.3389/fpubh.2017.00268

**Published:** 2017-09-29

**Authors:** Andrew E. Springer, Alexandra E. Evans, Jaquelin Ortuño, Deborah Salvo, Maria Teresa Varela Arévalo

**Affiliations:** ^1^Health Promotion and Behavioral Sciences, University of Texas Health Science Center at Houston School of Public Health-Austin, Michael & Susan Dell Center for Healthy Living, Austin, TX, United States; ^2^St. Edward’s University, Austin, TX, United States; ^3^Michael & Susan Dell Center for Healthy Living, University of Texas Health Science Center at Houston School of Public Health-Austin, Austin, TX, United States; ^4^Epidemiology, Human Genetics and Environmental Sciences, University of Texas School of Public Health-Austin/Michael & Susan Dell Center for Healthy Living, Austin, TX, United States; ^5^Center for Nutrition and Health Research, Instituto Nacional de Salud Pública, Cuernavaca, Mexico; ^6^Departamento de Ciencias Sociales, Pontificia Universidad Javeriana Cali, Cali, Colombia

**Keywords:** context, health and place, environments, settings, health promotion, health planning

## Abstract

The important influence of the environmental context on health and health behavior—which includes place, settings, and the multiple environments within place and settings—has directed health promotion planners from a focus solely on changing individuals, toward a focus on harnessing and changing context for individual and community health promotion. Health promotion planning frameworks such as Intervention Mapping provide helpful guidance in addressing various facets of the environmental context in health intervention design, including the environmental factors that influence a given health condition or behavior, environmental agents that can influence a population’s health, and environmental change methods. In further exploring how to harness the environmental context for health promotion, we examine in this paper the concept of *interweaving of health promotion into context*, defined as weaving or blending together health promotion strategies, practices, programs, and policies to fit within, complement, and build from existing settings and environments. Health promotion interweaving stems from current perspectives in health intervention planning, improvement science and complex systems thinking by guiding practitioners from a conceptualization of context as a backdrop to intervention, to one that recognizes context as integral to the intervention design and to the potential to directly influence health outcomes. In exploring the general approach of health promotion interweaving, we examine selected theoretical and practice-based *interweaving* concepts in relation to four key environments (*the policy environment, the information environment, the social/cultural/organizational environment*, and *the physical environment*), followed by evidence-based and practice-based examples of health promotion interweaving from the literature. Interweaving of health promotion into context is a common practice for health planners in designing health promotion interventions, yet one which merits further intentionality as a specific health promotion planning design approach.

## Introduction

A growing body of evidence highlights the influence of the context that surrounds us on our health and health-related behaviors, including where we live, study, work, pray, and play (1). Low-income populations who live in certain geographic areas of the United States (US), for example, have been found to live longer compared to populations of the same low-income status living in other parts of the country, with differences due not only to individual-level health behaviors and outcomes, such as physical activity (PA), smoking, and obesity, but also area-level characteristics, such as composition of educated population, immigrant population, and government expenditures (2). While selection bias is an important consideration for studies on place and health, the Moving to Opportunity study found that low-income families who were randomly assigned to live in economically better off census tracts experienced better health and social outcomes compared to families who remained in economically disadvantaged neighborhoods, such as lower rates of extreme obesity and diabetes (3) and increased college attendance and lower single parenthood for children moving at a younger age (4).

Beyond place of residence, specific organizational settings such as schools have also been found to influence health and health behavior. In examining PA engagement in the US, for example, some research finds that white adolescents are more physically active compared to other racial/ethnic groups (5, 6). However, when examining PA in relation to the schools that adolescents attend, white adolescent girls have been found to have the same levels, and white adolescent boys lower levels, of PA when compared to African-American and Hispanic adolescents attending the same economically disadvantaged schools (7). Childhood obesity also appears to pattern by the school the child attends. While white adolescents in the US tend to have lower levels of obesity compared to African-American and Hispanic adolescents (8, 9), our research with Texas public middle school students found similar levels of obesity among these three ethnic groups when attending the same high economically disadvantaged schools (10). Adjusting for the school a child attends has also been found to eliminate racial/ethnic disparities for a range of other health-related outcomes in research on fifth grade children from three large metropolitan areas in the US, including witnessing of violence, health status, and quality of life, as well as PA and obesity (11). These findings contribute to a growing body of evidence on the role of place [e.g., Ref. (12–15)] and settings [e.g., Ref. (16–19)] in shaping health and health behavior.

The important influence of the environmental context on health, which includes place, settings and the multiple environments within place and settings (e.g., policy, information, social/cultural/organizational, and physical environments) (20), has directed health promotion practitioners and researchers from a focus solely on changing individuals, as emphasized in earlier conceptualizations of the concept of health promotion (21), toward a focus on harnessing and changing context for the promotion of individual and community health. *Intervention Mapping (IM)* (22) is one of several health promotion planning frameworks [e.g., Ref. (23–25)] that explicitly guides planners in addressing environmental factors for health intervention planning. In addition to providing a robust model for health promotion planning that has been widely applied across a range of health issues, settings, and populations (22), IM directs health planners to identifying various facets of the environment that can be incorporated into health intervention design, including *environmental factors* that influence the health problem or risk behavior; *environmental agents* who can directly influence health and behavior and the environmental conditions that impact health and behavior; and *environmental change methods for influencing* different health determinants at different ecological levels, such as *organizational diagnosis and feedback, participatory problem solving*, and *advocacy*—methods aimed at changing organizational and societal level outcomes (22, 26).

In further exploring how to harness the environmental context for health promotion, we examine in this paper the concept of *interweaving* of health promotion into context, which focuses on designing health promotion interventions in concert with people’s settings and environments. *Health promotion interweaving* builds from current perspectives in health intervention planning (22, 26), improvement science, and complex systems thinking (27–31) by guiding practitioners from a conceptualization of context as a backdrop to intervention, to one that recognizes context as integral to the intervention design and to the potential to directly influence health outcomes. In exploring the general approach of health promotion interweaving, we examine selected theoretical and practice-based *interweaving* concepts in relation to four key environments (*the policy environment, the information environment, the social/cultural/organizational environment*, and *the physical environment*), and then illustrate health promotion interweaving with evidence-based and practice-based examples.

## *Interweaving*: Designing Interventions in Concert with Settings and Environments

Interweaving, defined as weaving or blending together (Webster Dictionary, 2017), is a common practice for health promotion planners in designing health promotion interventions in relation to people’s environmental context, yet one which we argue merits further intentionality as a specific health planning design approach and method. *Interweaving of health promotion into context* has also been described as *coupling* and *embedding* of health intervention with context (27–29) and generally refers to an intentional process of designing health promotion interventions, which may include health promotion strategies, practices, programs, and policies, to fit within, complement, and build from existing settings and environments. While concepts such as coupling and embedding hold specific relevance for harnessing context for health promotion (27–29), the concept of *interweaving* seeks to more directly communicate the idea of *blending together* the intervention with the properties of a given place or setting in order to create a unique and emergent health promoting context.

Health promotion interweaving into context embraces and aims to advance an *indigenous* health intervention development perspective in which interventions are developed from the “bottom up” in direct partnership with communities, building from site-specific knowledge, practices, and values (28, 32). This perspective differs from the dominant pipeline approach to knowledge generation in which health interventions are first designed by researchers under optimal conditions as part of an efficacy trial, and then tested under “real world” conditions as part of an effectiveness trial, where “real world” may be viewed as a potential for dilution of intervention effects, and “optimally designed” interventions often experience low diffusion (28, 32). In contrast to health intervention planning approaches that first begin with developing a program and then explore how the program fits within a given context, health promotion interweaving begins with identifying and understanding “real world” context, including the settings and multiple environments that surround people, which then become both the opportunity and platform for designing interventions. We posit that *health promotion interweaving* holds potential to not only enhance the population-specific relevance and sustainability of a given intervention, but also to increase impact on health outcomes by broadening the “environmental canvas” upon which to plan and build health interventions.

In guiding health promotion planners to more intentionally design health promotion interventions in relation to people’s environmental context, we recently published a basic environmental asset assessment conceptual framework (33) inspired in part by the IM needs assessment phase (22) and informed by ecological models of behavior (20, 34) and implementation science and systems thinking (27). Under this basic framework, health promotion practitioners are encouraged to first identify settings where to reach populations (e.g., school, worksite, church, community) and then explore assets of various environments (policy, information, social/cultural/organizational, and physical environments) within those settings that can be incorporated into health intervention design (33).

A settings and environments approach has been a central feature of other established health intervention planning processes and models (20, 34–37) and may provide additional benefits for health promotion planning. While ecological models are often defined in terms of levels (e.g., interpersonal, organizational, community, and societal levels) (38), conceptualizing the ecological space in terms of environments and behavioral settings (20, 33–37) offers further direction for health promotion planners as a given setting encompasses multiple environments and assets that can be activated for intervention design. For example, within a school setting, existing assets and environments that might be catalyzed for a given health promotion intervention include a parent/teacher organization within the school social/organizational environment, as well as the student morning announcements within the school information environment.

In building from this environmental asset assessment framework, we present in Table 1 selected theoretical and practice-based concepts that both support and provide further direction for *health promotion interweaving into context* as a health intervention design approach in relation to the *policy, information, social/cultural/organizational*, and *physical environments*. These theoretical and practice-based concepts were chosen to illustrate how health interventions can directly build from, connect with, and “interweave” health promotion into different environments located within settings and geographic place that hold potential to influence health and health behavior. In the following section, we describe these theoretical and practice-based *interweaving concepts* with examples from the health promotion literature and practice field.

**Table 1:** Exploring selected theoretical and practice-based concepts in support of health promotion interweaving into context as organized by key environments.

Concept	Definition	Practice or theory perspective
Interweaving (coupling, embedding)	The process of designing and inserting health promotion intervention into existing context, including settings and environments	*Complex Systems and Improvement Science* (27–31)

**Policy environment**		
Health-in-all policies	Incorporating health considerations into decision-making across sectors and policy areas with the aim of improving people’s health (39)	*Community and Municipal Planning*, NACCHO (40)

**Information environment**		
Environmental print	The print of everyday life, including the symbols, signs, numbers, and colors found in the school, neighborhood, and Internet (41). A concept from the field of childhood literacy that holds promise for enhancing everyday contexts for health communication	*Child Literacy*, Neumann (41)
Behavioral journalism	Incorporating authentic role model stories of behavior change into mass and local media based on priority population [(22) p. 393]	*Health Communication*, McAlister et al. (42), Reininger et al. (43)
Cues to action	Providing positive reinforcement for a health behavior or health action *via* visual cues (e.g., messages, symbols) and strategic placement of stimulus [(22) p. 381]	*Health Belief Model*, Janz and Becker (44)

**Social/cultural/organizational environment**		
Appropriable organization	Harnessing social organization that is created for one purpose to provide a valuable resource for other, different purposes (45)	*Social Capital Theory*, Coleman (45)
Mobilizing social networks and social support	“Encouraging social networks to provide informational, emotional, appraisal, and instrumental support.” [(26) p. 16]	*Theories of Social Networks and Social Support*, Holt-Lunstad and Uchino (46), Valente (47)
Structural redesign	Changing organizational elements such as mission, communication, reward systems, and job descriptions to support health promotion [(22) p. 395]	*Organizational Development Theory*, Cummings and Worley (48)
Common agenda	Creating a shared vision for change that includes a common understanding of the problem and joint approach to solving the problem through agreed-upon actions (49)	*Collective Impact*, Flood et al. (50)

**Physical environment and settings**		
Facilitation	“Creating an environment that makes the action easier or reduces barriers to action.” [(26) p. 6]	*Social Cognitive Theory*, Bandura (51)
Shared use	Establishing a formal or informal agreement between two or more separate entities, such as a school and a city or county, that describes the terms and conditions for shared use of public property or facilities (52)	*Community and Municipal Planning*, ChangeLab Solutions (52)

## Policy Environment

In harnessing the policy environment for health promotion, the *Health-in-All Policies* (HiAP) approach embodies the concept of health promotion interweaving. HiAP aims to improve population and individual-level health outcomes by incorporating health considerations into decision-making and policy areas across sectors of a community or society (39). In addition to exploring how a given policy or planning proposal may adversely impact health (53), HiAP explicitly promotes the incorporation of health promoting policies and actions in community planning through collaboration with non-traditional health partners, such as transportation, housing, land development, and employment-sectors that directly affect social determinants of health (39, 54). While HiAP is often considered at the community or societal levels—with a recent review indicating that HiAP is growing within municipal governments (55), HiAP represents a promising approach for harnessing the policy environment at different ecological levels, including the organizational and interpersonal levels such as worksites, schools, and even households. Examples of health promotion interweaving into the policy environment include the following:

- *Physical activity and city planning*: *Imagine Austin* is an example of a comprehensive long-term plan emerging in cities and counties across the US that incorporates quality of life and health considerations into city planning beyond just a focus on land use (56). With guidance from this plan, Austin residents voted in 2016 to approve a multi-million dollar bond package that includes funding for sidewalks, safe routes to school, bikeways, and urban trails (56).
- *Lactation support and the workplace*: State legislation in Texas under the Mother-Friendly Worksite Program from 1995 and more recent legislation under the Right to Express Breastmilk in the Workplace from 2015 directly interweaves health promotion into the worksite by providing recognition for and requiring that worksites in Texas support women to express milk at the workplace *via* a designated room or space as well as break time for lactation (57).
- *Teen driving and parent-imposed limits*: Simons-Morton et al.’s (58) findings on the reduction of risky driving among teenagers *via* the promotion of parent–teen driving agreements is an example of interweaving of health-related policies into the household setting that hold benefit for adolescent health promotion.

## Information Environment

The information environment exists in most behavioral settings (20) and can take on many forms, including written, symbolic, verbal, and non-verbal messaging (33). Promoting health *via* the information environment has been a key practice of health promotion practitioners, with examples that include inserting nutrition information in restaurant menus (59), installing seatbelt warning lights in the cars we drive (60), and delivering public service announcements *via* the television to promote parent–child communication about alcohol use (61). The concept of *environmental print*, which refers to the symbols, signs, numbers, and colors of everyday life that enhance children’s literacy (41, 62), holds relevance for harnessing the information environment by guiding health promotion practitioners to interweave health messaging across everyday-life contexts of individuals. Examples of theoretical change methods cited in IM (22) that can contribute to the creation of an environmental print for health include *cues to action* (44), in which cues are embedded into a given behavioral setting, such as stickers within bathrooms to promote handwashing with soap (63) and point-of-choice prompts that encourage stair climbing (64), and *behavioral journalism*, in which health-related role model stories are interwoven into newspapers, magazine articles, and other media (42, 43). Examples of interweaving of health promotion into everyday information environments include:

- *Healthy eating and PA promotion on the Texas–Mexico border*: In promoting fruit and vegetable (FV) consumption and PA among a US–Mexico border population living in one of the poorest counties in the US, role model stories and personal testimonies were delivered *via* 30-s radio segments on Spanish-language stations during morning drive times, 4–5 min weekly TV health segments shown during a Spanish-language morning show, and a Spanish-language newsletter and website (65). Participants exposed to both radio and TV messages consumed more portions of FV, and participants exposed to radio and Community Health Worker discussions were more likely to meet PA recommendations (65).
- *Substance use prevention in secondary school students*: In harnessing the school and community information environments for adolescent substance use prevention, Slater and colleagues (66) inserted print messages into posters, book covers, tray liners, T-shirts, water bottles, rulers, and lanyards within US school settings as well as verbal messaging such as public service announcements delivered *via* community organizations. At two-year follow-up, youth in the eight media-enhanced intervention communities reported lower marijuana and alcohol use compared to a classroom curriculum comparison condition (66).

## Social/Cultural/Organizational Environment

With foundation in ecological models of health behavior (20, 34), we broadly define this environment in terms of the social and cultural organization that exist within a given setting, as well as the social, cultural, or organizational factors that relate to health and health behavior (33). Three theoretical concepts for interweaving health promotion into this environment are *appropriable organization* from Social Capital Theory (45); *mobilizing social networks* and *social support*—theoretical methods identified in IM (26) and based in theories of the same names (46, 47); and *structural redesign*, also cited in IM (22) and stemming from theories of organizational development (48) [see also *normative restructuring* (29)]. These concepts embrace an interweaving approach *via* appropriating, mobilizing, and restructuring existing social organization (e.g., a school committee or worksite) or elements of an organization (e.g., organizational norms, mission, roles) that were created for one purpose to be activated for health promotion purposes. As interweaving into existing organization holds important ethical implications, the creation of a *common agenda*—a concept from the field of Collective Impact in which two or more parties create an agreed-upon approach for action (49)—merits emphasis. Examples of interweaving health promotion into the social/cultural/organizational environment include:

- *Contraceptive use and drug shops in Uganda*: In response to the low accessibility of contraceptives in rural and peri-urban areas of Uganda, the STRIDES for Family Health project successfully incorporated family planning products and services into private drug shops, which are similar to pharmacies but not required to employ trained pharmacists (67). Drug shop operators in four districts were trained to counsel clients, of whom over half were of low socioeconomic status, and safely administer contraceptive injections, resulting in high levels of client satisfaction and delivering equivalent proportions of contraceptive protection compared to clinics and community health workers (67).
- *HIV risk reduction and peers*: Activating peer networks and peer-led education have been found to be an effective strategy in promoting HIV risk reduction behaviors [e.g., Ref. (68–70)] and HIV screening (71) among diverse at-risk populations. This large and growing body of research underscores both the power and potential of mobilizing existing social networks within the social environment for health promotion.
- *Physical activity promotion and the school setting*: Marathon Kids (MK), an international non-profit organization, interweaves PA into existing school schedules and organization by encouraging teachers to incorporate opportunities for children to run during recess and other times of the day, tracking miles run as part of classroom learning on topics such as math, and inserting MK awards into existing end-of-year school award ceremonies to provide positive PA reinforcement, among other school organizational enhancements (72). Children attending economically disadvantaged schools that participated in MK reported increased PA participation and other related outcomes such as increased athletic identity self-concept (72).

## Physical Environment

The physical environment, defined broadly as features of the built and natural environments (73), has received increased attention in the past several years for its effects on health (74), including physical health (75–79), mental health (80–83), and social health (84, 85). While the growing body of evidence on the built and natural environment and health is beyond the scope of this paper [see Ref. (74, 79, 83, 86)], this literature underscores the importance of how we design, shape, organize, and connect with the physical environment in enhancing population health. Two basic *interweaving* concepts that provide direction for harnessing the physical environment for health promotion are *facilitation* and *shared use*. With applicability across the environments presented above, facilitation is a broad and robust theoretical method (22) with roots in Social Cognitive Theory (51) that refers to creating an environment that makes a given health action easier. A shared use agreement, a specific example of how facilitation can be operationalized for the physical environment, is an important practice-based concept in which an agreement is made between two or more parties to allow use of a given physical space for health-related activity (52). Examples of interweaving health promotion into existing physical environments include:

- *Pop-up parks, PA, and social capital*: Pop-up parks, like Ciclovias/Recreovias (open-street events) (87, 88) or dual-use facilities (e.g., parks located in-school grounds that are open to the community after school hours) (89) challenge the notion that urban spaces are naturally permanent. Pop-up parks are by definition small and temporary, and are located in areas typically reserved for cars (parking lots, streets) (90). A recent study in Los Altos, CA, USA, found that a small, urban pop-up park attracted a large number of multigenerational users (91). High levels of PA in the pop-up park were observed among youth. Among users, the presence of the pop-up park was associated with less screen time, and with more time spent at a park, outdoors, and in the downtown central business district of Los Altos (91). Pop-up parks represent a promising strategy for communities with limited space for public recreation facilities, both to promote PA, as well as to improve social capital and quality of life through the revitalization of urban settings.
- *Train stations and blood pressure screening*: In 2016, the St. Louis County Department of Public Health and a non-profit arm of the Bi-State Development Agency received federal funding to provide preventative health-care services such as blood pressure screening for commuters at a train station in north St. Louis County, Missouri, USA (92). This innovative project demonstrates the potential of interweaving health promotion into existing physical public spaces such as train stations that have wide population reach.

## Applying Health Promotion Interweaving to Health Promotion Planning

A *health promotion interweaving into context* approach, as illustrated with the above examples, holds important implications as a specific design strategy for health promotion planning frameworks such as IM. Step 1 of IM, which includes conducting a health needs assessment (22), presents an ideal opportunity for incorporating health promotion interweaving by directing planners to identify not only the factors contributing to the health problem, but also the environmental assets that can be incorporated into the design of the health intervention. Examples of environmental assets that may be identified during the needs assessment phase include a code of conduct for employees in the *policy environment (interweaving concept: HiAP)*, existing communication channels such as a parent bulletin board in the school *information environment (environmental print)*, established forms of social organization such as a neighborhood civic council in the *social/cultural/organizational environment (appropriable organization)*, and existing physical spaces for intervention activities in the *physical environment (shared use)*. A health promotion interweaving approach can also inform other health promotion planning steps as described in IM, including the identification of *environmental outcomes* of a given intervention (Step 2 of IM) (e.g., a HiAP approach that includes a policy on prohibiting e-cigarette use delivered *via* student handbooks), the provision of *a platform* or *practical application for delivery of key theoretical methods* (Step 3) (e.g., role modeling stories on sleep health delivered *via* the company newsletter), guidance with the overall *design of a given health intervention* (Step 4) (e.g., the four environments described above inform the creation of program components), and identification of existing human resources *who can implement* a given aspect of the intervention (Step 5) (e.g., cafeteria workers encourage students to take a fruit or vegetable in the cafeteria line). Finally, the health promotion interweaving concepts presented in Table 1 aim to advance an approach of “designing from within” a given setting or system, in order that a given health intervention “sticks” and does not wash out over time, an ongoing challenge of health interventions (27).

## Discussion

Health promotion planning frameworks such as IM (22) provide helpful guidance in developing health interventions focused not only on changing individuals, but also the environmental context with which the individual interacts on a daily basis. In this paper, we aimed to complement such planning frameworks by examining how health promotion planners can intentionally design health promotion interventions that build from a priority population’s context *via* the concept of *health promotion interweaving*. A specific contribution of this paper is the exploration of theoretical and practice-based interweaving-related concepts that provide guidance for designing health interventions in relation to environments and settings. Given the potential to directly harness contexts that can shape health and health behavior while broadening the environmental canvas for developing health promotion strategies, health promotion interweaving-related approaches merit greater emphasis and study within the field of health promotion intervention planning.

In recent years, a growing number of organizations and initiatives have embraced similar *health-by-design* approaches in which interventions are explicitly developed in relation to the environmental context, including the *Health, Behavioral Design, and Built Environment Project* of the National Collaborative on Childhood Obesity Research (93), OLE! Texas- a state-wide initiative aimed at enhancing childcare outdoor environments (94), the Michael & Susan Dell Foundation funded *Go Austin-Vamos Austin* place-based initiatives (95), and the Robert Wood Johnson Foundation’s built environment initiatives (96), among others. While the selected examples provided in this paper aim to both illustrate and provide foundation for the practice of interweaving of health promotion into context, we recognize that more applied research and practice is needed to fully understand and harness the power of interweaving. In looking forward, a growing body of research on concepts from complex systems science and social-ecological theory offer promising direction for exploring further how health promotion is interwoven across a given setting and how different environments may interact with each other over time to produce (or inhibit) health and health behavior change. These concepts include: *extensiveness and intensiveness* of an intervention within a given setting (27)—which may include assessing how a given intervention is interwoven across environments of a given setting, *interactions of influence* across environments and levels that may enhance or inhibit behavior change (34), *emergent properties* within a setting that result from a given modification or intervention (28, 29)—with implications that include understanding how health interventions that are interwoven into environments produce changes—possibly positive or negative—across a given setting or system, *timing and sequencing* of intervention modifications within settings (28, 29, 93)—recognizing that the impact of health promotion interweaving on health or health outcomes may not follow a linear sequence, and *agency* for intervention (28, 29, 93), among others [see Ref. (28, 29) for recent reviews].

As the concept of health promotion interweaving is context specific by nature, new approaches may be needed for exploring how to best evaluate such interventions. The complex systems literature holds relevance for exploring how a health promotion interweaving approach may best be evaluated (28, 29, 31). Two relevant concepts for evaluation of health promotion interweaving approaches from this literature are the need for a *new conceptualization of fidelity of program implementation* (27), and the need for a shift from a linear planning and evaluation framework to one that *acknowledges the complexity of context* and the *importance of process* (28, 29, 31). Fidelity under a traditional program evaluation would focus on keeping constant the delivery of the intervention across different study sites, yet this conceptualization of fidelity contradicts the nature of health promotion interweaving, which embraces the uniqueness of different settings as an opportunity for identifying setting-specific intervention approaches *via* harnessing its multiple environments (policy, information, social/organizational, and physical). In recognition of the diversity of sites and systems, Hawe et al. recommend a refined conceptualization of fidelity—one that conceives the intervention as a dynamic event within a system where the “function” of a given intervention (e.g., delivering 30 min of PA *via* a defined process for identifying strategies) is more important than the form (e.g., strategies for delivering PA are tailored to a given school), which may vary by site (27). Related to this new conceptualization of fidelity is the need to identify evaluation approaches that move from a linear process, often based on an oversimplified linear logic model in which the planner is expected to have all the answers upon intervening in a given system (28), to one which embraces complexity of context and emphasizes a process of co-learning with people from a given context to identify environmental modifications and make adaptations to these approaches over time (28, 29). Given that public health actions and effects often do not follow a linear time period and may require long periods of time (28, 29, 93), Rutter and colleagues emphasize the importance of tracking proximal, intermediate, and distal processes and outcomes, as well as the importance of modifying approaches in responses to changes in systems (31). The growing field of participatory learning and action (97, 98)—which includes visual and group-based inquiry methods for exploring the process of a given intervention (99, 100)—holds specific relevance for engaging populations in co-learning around implementation and evaluation of health promoting interweaving approaches.

While we were purposeful in this paper in exploring interweaving as a practical health promotion planning approach for health promotion practitioners—building from Einstein’s famous dictum that “everything should be made as simple as possible, but not simpler,” we also recognize the need for field-based research to inform best approaches for interweaving and understand further its impact on health promotion. Kok and colleagues (26), the authors of IM, have provided important advances to the field of health promotion planning in recent years by examining the parameters, or conditions, under which a given environmental change method may be effective. While health promotion interweaving can inform the approach for designing interventions in relation to environments, and the different environments cited in this paper may help provide the “canvas” upon which to design interventions, there is a need to understand the parameters that make health promotion interweaving an effective health promotion planning approach that ultimately results in positive individual-level and community-level health changes. As an example, and as mentioned above, “appropriating” organization may hold important ethical implications, and as such, a possible parameter for health promotion interweaving is that it is most effective when people from a given setting are involved in making decisions regarding health promotion changes to their organization. Furthermore, we provided only a selected sample of interweaving-related concepts in this paper to illustrate the overall interweaving design approach; the growing menu of theoretical methods identified *via* approaches such as IM (22, 26) offers further direction for exploring best approaches for interweaving.

We also recognize other inherent limitations of our basic overview of interweaving in this paper, including the challenge of classifying specific environments, given the overlap of different types of environments such as a “policy environment” and a “social/organizational environment,” as well as our limited conceptualization of the environment in terms of the four key environments examined. As we previously recognized (33), there are undoubtedly other important environments that should be explored that have relevance for shaping health and health behavior. One such environment is the *economic environment*, which Swinburn and colleagues define in their ANGELO obesogenic environmental planning framework in terms of costs related to food and PA (35). While economic forces often appear outside the control of health promotion practitioners, Swinburn and colleagues (35) offer examples of how the economic environment may be harnessed for health promotion *via* monetary incentives in terms of taxes, pricing policies, and subsidies; financial support for health promotion programs; healthy food purchasing policies; and budget allocations for a given health intervention such as the creation of a bike path. Economic subsidies, in particular, represent a potential interweaving concept by directly shaping the economic environment of a given setting or organization to be more supportive of a given health behavior or outcome. In central Texas, for example, the non-profit organization Sustainable Food Center (SFC) established a “double dollar” program for their farmers’ markets in which economically disadvantaged residents can double the amount of government subsidies (e.g., SNAP, WIC, and FMNP) to increase their purchasing power of healthy foods at SFC markets (101). Beyond directly intervening in the economic environment, the interweaving concepts and other environments explored in this paper also hold relevance for enhancing health for economically disadvantaged populations, as illustrated by several of the examples cited in this paper that included low-income populations. While further exploration of each of these environments, as well as additional environments such as the economic environment, are warranted, the basic settings and environments framework that we present here provides an initial foundation to build upon in exploring health promotion interweaving.

Health promotion interweaving presents a basic yet promising approach for designing health promotion interventions that moves beyond a perspective of context as the delivery setting of an already-designed intervention, to one that embraces context as integral to the design of health interventions. We look forward to continuing to co-learn with health promotion practitioners and researchers about best practices for interweaving health promotion into context.

## Ethical Considerations

This paper is based on a review of existing literature and does not report any individual data.

## Author Contributions

AS and AE jointly conceived of this paper. AS drafted the paper with major section contributions and editing of the manuscript from AE and DS. JO and MA provided key contributions with review of the literature and editing of the paper.

## Conflict of Interest Statement

The authors declare that the research was conducted in the absence of any commercial or financial relationships that could be construed as a potential conflict of interest. The reviewer SK and the handling editor declared their shared affiliation, and the handling editor states that the process nevertheless met the standards of a fair and objective review.
